# The Acute Effects of Amyloid-Beta_1–42_ on Glutamatergic Receptor and Transporter Expression in the Mouse Hippocampus

**DOI:** 10.3389/fnins.2019.01427

**Published:** 2020-01-17

**Authors:** Jason H. Y. Yeung, Thulani H. Palpagama, Warren P. Tate, Katie Peppercorn, Henry J. Waldvogel, Richard L. M. Faull, Andrea Kwakowsky

**Affiliations:** ^1^Centre for Brain Research, Department of Anatomy and Medical Imaging, Faculty of Medical and Health Sciences, The University of Auckland, Auckland, New Zealand; ^2^Department of Biochemistry, School of Biomedical Sciences, University of Otago, Dunedin, New Zealand

**Keywords:** amyloid beta, glutamate receptor, glutamate transporter, hippocampus, Alzheimer’s disease

## Abstract

Alzheimer’s disease (AD) is the leading type of dementia worldwide. Despite an increasing burden of disease due to a rapidly aging population, there is still a lack of complete understanding of the precise pathological mechanisms which drive its progression. Glutamate is the main excitatory neurotransmitter in the brain and plays an essential role in the normal function and excitability of neuronal networks. While previous studies have shown alterations in the function of the glutamatergic system in AD, the underlying etiology of beta amyloid (Aβ_1–42_) induced changes has not been explored. Here we have investigated the acute effects of stereotaxic hippocampal Aβ_1–42_ injection on specific glutamatergic receptors and transporters in the mouse hippocampus, using immunohistochemistry and confocal microscopy 3 days after Aβ_1–42_ injection in aged male C57BL/6 mice, before the onset of neuronal cell death. We show that acute injection of Aβ_1–42_ is sufficient to induce cognitive deficits 3 days post-injection. We also report no significant changes in glutamate receptor subunits GluA1, GluA2, VGluT1, and VGluT2 in response to acute injection of Aβ_1–42_ when compared with the ACSF-vehicle injected mice. However, we observed increased expression in the DG hilus and ventral stratum (str.) granulosum, CA3 str. radiatum and str. oriens, and CA1 str. radiatum of the GluN1 subunit, and increased expression within the CA3 str. radiatum and decreased expression within the DG str. granulosum of the GluN2A subunit in Aβ_1–42_ injected mice compared to NC, and a similar trend observed when compared to ACSF-injected mice. We also observed alterations in expression patterns of glutamatergic receptor subunits and transporters within specific layers of hippocampal subregions in response to a microinjection stimulus. These findings indicate that the pathological alterations in the glutamatergic system observed in AD are likely to be partially a result of both acute and chronic exposure to Aβ_1–42_ and implies a much more complex circuit mechanism associated with glutamatergic dysfunction than simply glutamate-mediated excitotoxic neuronal death.

## Introduction

Beta amyloid (Aβ) is a ∼4 kDa peptide product derived from the cleavage of amyloid precursor protein (APP). In normal physiology, the APP molecule can be cleaved by two different secretases; cleavage by alpha and gamma secretase yields non-neurotoxic fragments (Sadigh-Eteghad et al., 2015), while Aβ is generated from the cleavage of APP through the beta and gamma secretase pathway (Selkoe, 1998). This Aβ can further aggregate into larger polymeric structures, including oligomers, protofibrils, and amyloid fibrils, each of which exhibit different functional properties (Finder and Glockshuber, 2007). Amyloid plaques are formed from the assembly of insoluble amyloid fibrils, whereas amyloid oligomers are soluble and appear to exhibit much higher cytotoxicity, perhaps due to their soluble nature (Dahlgren et al., 2002). Both amyloid plaques and soluble amyloid oligomers have been implicated in the pathogenesis of Alzheimer’s disease (AD).

AD is a major neurodegenerative disorder characterized by the presence and accumulation of two pathological hallmarks: (Aβ) aggregates and neurofibrillary tau (Glenner and Wong, 1984; Grundke-Iqbal et al., 1986). The amyloid cascade hypothesis is one of the earliest and leading hypotheses in relation to both the initiation and progression of AD. There is contention as to which form of Aβ is responsible for the pathophysiological responses seen in AD, although current data point toward smaller soluble Aβ oligomers as playing the most critical role, with amyloid plaques contributing to but not essential in the pathogenesis of AD (Sakono and Zako, 2010).

Glutamate comprises a major excitatory system within the CNS, and has a critical role in a variety of homeostatic and neurological processes. It acts on a variety of receptors, broadly categorized as ionotropic and metabotropic. Ionotropic receptors include the *N*-methyl-D-aspartate receptor (NMDAR), alpha-amino-3-hydroxy-5-methyl-isoxazolepropinoic acid receptor (AMPAR), and kainate receptor classes. The metabotropic class of receptors are subdivided into three functionally distinct groups; group I are coupled with phospholipase C, while group II and III are coupled with adenylyl cyclase (Kew and Kemp, 2005). While ionotropic receptors are present predominantly on the post-synaptic membrane, metabotropic receptors have been found to be expressed on both neuronal and glial cells (Niswender and Conn, 2010). The differential spatial localization of these two receptor subtypes appear to facilitate differential activation of receptors in proportion to the amount of glutamate released from the presynaptic space (Nusser et al., 1994). Vesicular glutamate receptors (VGluTs), categorized into VGluT1 and VGluT2, are present at presynaptic neurons and are vital in maintaining vesicular glutamate concentrations (Fremeau et al., 2001).

The glutamatergic system has also been heavily implicated in the pathogenesis of AD, however the relationship between Aβ and glutamatergic dysfunction is still not well understood. Studies have shown associations between glutamatergic dysfunction and Aβ exposure, with Aβ exposure associated with the endocytosis of NMDARs and AMPARs (Snyder et al., 2005; Hsieh et al., 2006). Although changes in expression of components of the glutamatergic system in AD have been noted in previous studies, there has been little examination on whether this is due to direct Aβ interaction, secondary to downstream effects, or associated with other pathways altogether.

The importance of investigating acute changes lies in the possibility of early phenomena not captured in later stages of Aβ interaction. Such physiological changes have been observed in human patients, with an increase in glutamatergic synapses observed in mildly cognitively impaired patients and subsequent reduction in AD patients, potentially reflecting a compensatory mechanism (Bell et al., 2007). Intracerebroventricular injection of Aβ oligomers in rats has been associated with memory deficits and cholinergic neuron loss in the acute setting. Acute exposure of rats to Aβ oligomers for 1, 3, 7, 21 days has shown pathophysiological alterations, including delayed increase in activated microglia and a decreased cholinergic neuronal number observed at day 21 (Wong et al., 2016). There are a limited number of studies that have examined acute Aβ_1–42_ -induced behavioral deficits (Kim et al., 2016; Kasza et al., 2017). Therefore, we have performed a thorough behavioral examination 3 days post-Aβ_1–42_ injection. Furthermore, there have not yet been studies examining acute effects of Aβ exposure on glutamatergic function. This is the first comprehensive anatomical study to characterize the subregion- and cell layer-specific effect of acute Aβ_1–42_ administration on the expression of specific glutamate receptors and transporters in the mouse hippocampus.

## Materials and Methods

### Aβ_1–42_ Preparation

**FIGURE 1:**
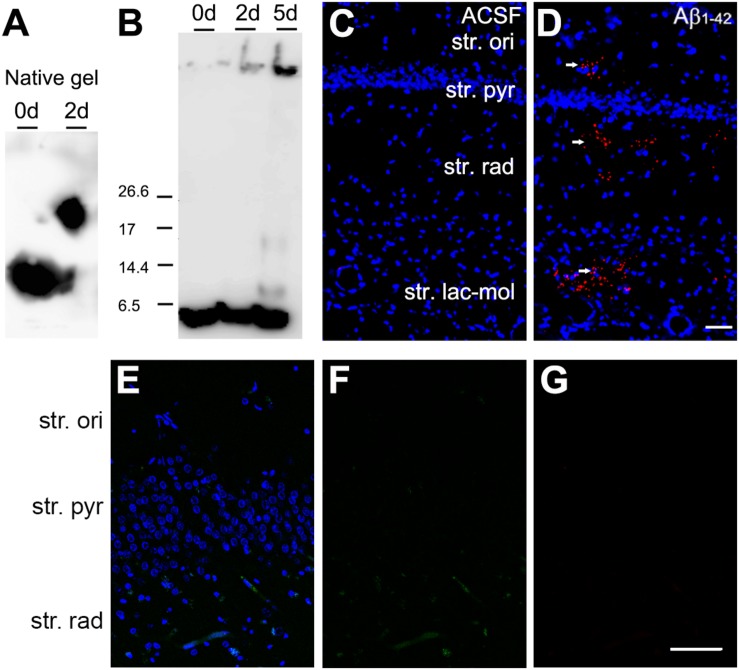
Aging of Aβ_1–42_, and detection at the injection site with Aβ immunolabeling. **(A)** Western Blot of a non-dissociating gel after aging Aβ_1–42_ for 48 h. The Aβ_1–42_ monomer, and possible aggregates were detected with antibody 4G8. **(B)** Western blot of an SDS dissociating gel after aging Aβ_1–42_ for up to 120 h. Bands detected with antibody 4G8 can be seen at the sizes of monomer, dimer and tetramer and a higher order aggregate. **(C,D)** Aβ_1–42_ immunolabeling (red on **D**) in the CA1 region of the mouse hippocampus after Aβ_1–42_ injection [**(C,D)** Hoechst (blue); **(D)** Aβ_1–42_ (red)]. Scale bar, 50 = μm. **(E,F)** The omission of the primary antibodies resulted in complete absence of the immunoreactivity [**(E)** Hoechst (blue), goat anti-rabbit Alexa Fluor 488 (green), goat anti-guinea pig Alexa Fluor 647 (red); **(F)** goat anti-rabbit Alexa Fluor 488 (green); **(G)** goat anti-guinea pig Alexa Fluor 647 (red)]. Scale bar: 70 = μm.

Method for preparation of Aβ_1–42_ is as described in Wilson C. M.Sc. Thesis, University of Otago (2007) and Kwakowsky et al. (2016). In short, Aβ_1–42_ is produced as a recombinant protein fused with maltose binding protein (MBP) in *Escherichia coli*. This strategy utilizes the solubilizing character of the MBP (product of the MalE gene) to ensure expression of soluble protein at high concentration (Kapust and Waugh, 1999). After expression in bacteria, the product was purified on an amylose column to which the MBP segment of the protein binds. Following binding to amylose resin, the pure fusion protein was eluted from the resin with maltose and concentrated by ammonium sulfate precipitation. Carrier MBP was cleaved off the fusion protein by Factor X protease, and the released Aβ_1–42_ isolated and further purified by hydrophobic chromatography with 0–50% v/v acetonitrile/0.1% v/v TFA, using FPLC. Fractions containing pure Aβ_1–42_ were detected immunologically with an antibody against residues 17–24 of Aβ_1–42_ and lyophilized to remove solvent. Mass spectrometry confirmed the expected molecular ion for the desired product. Prior to stereotaxic intrahippocampal injection, Aβ_1–42_ was dissolved in Artificial cerebrospinal fluid (ACSF) and ‘aged’ at 37°C for 48 h to facilitate the formation of toxic soluble aggregates. The optimal incubation time for preparations of Aβ_1–42_ to produce the highly toxic oligomers varies from preparation to preparation but is generally is 48–120 h. Western blots of an aging profile of Aβ_1–42_ are shown in Figure 1, analyzed both on non-dissociating gels (A), where the monomer decreases and an oligomer appears by 48 h, and SDS gels (B) where the dimer and trimer of Aβ_1–42_ are seen at 5 days as well as a higher molecular weight oligomer. Only aggregates from misfolded Aβ_1–42_ are deduced to be SDS insoluble (Hillen, 2019), explaining why SDS gels show lower amounts with the less stably-aggregated species dissociated by the SDS. Following gel electrophoresis of Aβ_1–42_ samples on a 12.5% acrylamide gel run under non-dissociating conditions, or a 16% acrylamide ‘peptide’ (Kolby) gel under dissociating conditions with SDS, samples were transferred to a PVDF membrane at 100 V for 1 h. After brief staining with Ponceau red to mark peptide markers, the PVDF was immersed with rocking for 2 h at room temperature (RT) in 1% milk powder in Tris-buffered saline, 0.1% Tween (TBS-T) (blocking solution), and then with primary antibody, 4G8 diluted in blocking solution, overnight at 4°C. Following washing 3 min × 10 min with TBS-T the blot was incubated with secondary antibody (1:5000 anti mouse HRP in TBS-T) at RT 1 h with rocking. After wash 3 min × 10 min with TBS-T signals were developed with the ECI reagent.

### Animals and Tissue Preparation

All experiments were approved and performed in accordance with the regulations of the University of Otago and the University of Auckland Animal Ethics Committees. Mice were housed under standard laboratory conditions and maintained in a 12 h light–dark cycle at the Hercus Taieri Resource Unit, University of Otago and Vernon Jensen Unit, the University of Auckland with food and water ad lib. Prior to surgery, 18 months old C57BL/6 male mice were anesthetized by subcutaneous injection of 75 mg/kg ketamine and 1 mg/kg domitor. Bilateral hippocampal stereotaxic surgery was performed, with coordinates for injection determined relative to the bregma (anterior–posterior, −2.0 mm; medial-lateral, ±1.3 mm; dorsal-ventral, −2.2 mm) with 1 μL 20 μM aggregated Aβ_1–42_ or ACSF injected at a speed of 0.1 μl/min. Following surgery, 1 mg/kg antisedan was subcutaneously administered to reverse anesthesia. Naïve control (NC) animals did not undergo any surgical procedures. Mice used in this study were categorized into three groups: NC (*n* = 6), ACSF-injected (*n* = 12), and Aβ_1–42_-injected (*n* = 12).

After 3 days post-Aβ_1–42_ injection, animals were deeply anesthetized via overdose of ketamine and domitor and perfused transcardially with 20 mL of ice-cold 4% paraformaldehyde in phosphate buffer (pH 7.6). For the Aβ_1–42_ immunohistochemistry experiment animals were perfused transcardially after 90 min of Aβ_1–42_ injection. Brains were removed and post-fixed in paraformaldehyde solution for 2 h at RT and then incubated in 30% sucrose in Tris-buffered saline (TBS) solution overnight at 4°C. Four sets of 30-μm thick coronal brain sections were cut using a freezing microtome. The sections were then stored in antifreeze solution at −20°C until use.

### Fluorescent Immunohistochemistry

Free-floating fluorescent immunohistochemistry was used to examine the expression of glutamate receptors AMPAR, NMDAR, and glutamate transporters VGluT1, VGluT2. Immunohistochemistry was performed as described by Kwakowsky et al. (2016). Tissue sections were blocked using 1% (v/v) goat serum in 0.05M tris buffered saline (TBS)/0.3% v/v Triton/0.25% w/v BSA (TTB) for 1 h at RT. Sections were then washed in TBS for 3 min × 10 min and incubated for 72 h in primary antibody specific for glutamate receptors and transporters at 4°C (Table 1). Specificity of the primary antibodies has been tested using western blotting and reported previously for each of the antibodies GluA1 (Zhu et al., 2017; Song et al., 2019), GluA2 (Banerjee et al., 2013; Hussain and Davanger, 2015), GluN1 (Morimura et al., 2017; Seigneur and Südhof, 2018), GluN2A (Atkin et al., 2015; Konstantoudaki et al., 2016), VGluT1 (Venniro et al., 2017; Nakano et al., 2018), VGluT2 (Hernández et al., 2015; Nakano et al., 2018), and Aβ_1–42_ (Kwakowsky et al., 2016) (Figures 1A–D). Following 3 min × 10 min washes in TBS, the sections were incubated at RT for 1 h in secondary antibodies goat anti-mouse Alexa Fluor 647 (1:500, A21236, Thermo Fisher, Waltham, MA, United States), goat anti-rabbit Alexa Fluor 488 (1:500, A11034, Thermo Fisher), and goat anti-guinea pig Alexa Fluor 594 (1:500, A11076, Thermo Fisher) diluted in TTB. Sections were then washed in 3 min × 10 min TBS prior to 15 min RT incubation of Hoechst nuclei counterstain (1:10000, H3570 Thermo Fisher) diluted in TTB followed by 3 min × 10 min TB wash. Sections with the primary antibody omitted were run in tandem with each experiment. The omission of the primary antibodies resulted in complete absence of the immunoreactivity (Figures 1E,F). Sections were mounted in gelatin, air dried overnight at RT, rehydrated, cover slipped with Mowiol mounting medium, and sealed with nail varnish.

**TABLE 1:** Primary antibodies used in this study.

**Immunogen**	**Source, host, species, catalog number**	**Dilutions**
KLH-conjugated linear peptide corresponding to human glutamate receptor 1 at the cytoplasmic domain	Millipore, Rabbit, AB-1504	1:200
Peptide fragment corresponding to amino acid residues of rat AMPA receptor 2	Alamone, Rabbit, AGC-005	1:500
Recombinant protein corresponding to AA 660 to 811 from rat GluN1	Synaptic Systems, Mouse, 114-011	1:200
Peptide GHSHDVTERELRN(C), corresponding to amino acid residues 41–53 of rat NMDA Receptor 2A	Alamone, Rabbit, AGC-002	1:500
Amino acid segment from C-terminal of mouse VGluT1 protein	Frontier Institute, Guinea Pig, VGluT-GP-Af570	1:200
559–582 amino acid segment from C-terminal of mouse VGluT2	Frontier Institute, Guinea Pig, VGluT-GP-Af810	1:1000
Whole Aβ_1–42_ peptide	Thermo Fisher Scientific, Rabbit, PA3-16761	1:500
Peptide corresponding to amino acid residues 17–24 of Aβ_1–42_ (4G8).	Sigma, Mouse, A1349	1:300

### Behavioral Testing

Behavioral testing was performed to elucidate the effects of Aβ_1–42_ on the cognitive performance of the mice using behavioral tests that target different types of hippocampal-dependent memories, including long-term spatial memory [novel object alteration (NOALT) and novel object recognition test (NORT)], as well as non-spatial memory (passive avoidance test). The NOALT and NORT behavioral tests were started at 9 AM and the passive avoidance test 11 AM, and behavioral analysis was performed using the tracking image analyzer system EthoVision XT 9 (Noldus).

#### Novel Object Alteration Test (NOALT)

The NOALT test was performed in a square arena that was surrounded by non-transparent plexiglass walls (25 cm × 29 cm × 25 cm). Each mouse was placed in the arena individually and given 10 min to habituate to the environment. Next, two identical objects were introduced in the arena at designated locations, and the mice were given 5 min to interact with and explore the objects. The following day (24 h later), one of the identical objects was placed in a new location, and the behavior of the mice was recorded over a 5 min testing period. The testing apparatus was cleaned between animals with 5% acetic acid to minimize olfactory cues. The discrimination ratio (DR) for a novel over a familiar object was calculated as follows: time spent near the object at the new position minus the time spent near the object at the old position, divided by time spent near the object at the new position plus the time spent near the object at the old position.

#### Novel Object Recognition Test (NORT)

Novel object recognition test was performed in the same arena as the NOALT. Animals were allowed to explore a set of two identical objects for a 10 min period, afterward the mice were returned to their cages. The next day (24 h later) the animals were presented with a similar set of objects but one object was novel to them; they were allowed to freely explore the objects again for a 5 min period. The amount of time spent to explore the new object is considered as an index of recognition memory. The DR for a novel over a familiar object was calculated as follows: time near a new object minus the time near the old object, divided by time near the new object plus the time near the old object (Kwakowsky et al., 2016).

#### Passive Avoidance Test

The passive avoidance test was performed following the NOALT or NORT. This associative learning task was conducted in a two-compartment box made of one bright compartment and one dark compartment (16 cm × 18 cm). During habituation, the mouse was placed in the bright compartment, and the mouse gained access to the dark compartment. When the mouse entered the dark compartment the door was closed, and the mouse was briefly administered a 0.3-mA electric shock on the foot for 2 s as an aversive stimulus. After 30 s the animal was returned to its home cage. Three hours later, the animal was returned to the bright compartment with the sliding door open. The animal now had the option to avoid or enter the dark compartment. The latency period before the mouse entered the dark compartment was measured.

### Imaging and Analysis

Imaging was conducted using a Zeiss 710 confocal laser-scanning microscope (Carl Zeiss, Jena, Germany). Regions and layers were differentiated based on cell type and relative location, utilizing Hoechst staining. Integrated density measurements were undertaken using ImageJ. The size of the measured areas as follows: 21,352 μm^2^ for the CA1 region, 4,761 μm^2^ for the CA3 region, and 12,295 μm^2^ for the DG. Specifically, intensity measurements were taken in the regions of the stratum (str.) pyramidale, str. radiatum and str. moleculare of the CA1 and CA3 regions, and the hilus, str. moleculare, and str. granulosum of the DG. The experimenter was blinded to avoid any potential bias during image acquisition and analysis.

### Statistical Analysis

Data in all experiments are expressed as mean ± SEM. To examine the differences between groups, a Kruskal–Wallis test was conducted for the data obtained, using Graph-Pad Prism software (GraphPad Software, San Diego, CA, United States; RRID:SCR_002798) with a *p*-value of *p* < 0.05 considered significant, as the data did not meet the assumptions of parametric tests assessed by the D’Agostino-Pearson omnibus and Brown-Forsythe tests. Adobe Photoshop CC 2017 (Adobe Systems Software, San Jose, CA, United States) was used to prepare the figures.

## Results

### Expression of AMPA Receptor Subunits in the Hippocampal CA1, CA3, and Dentate Gyrus Regions

**FIGURE 2:**
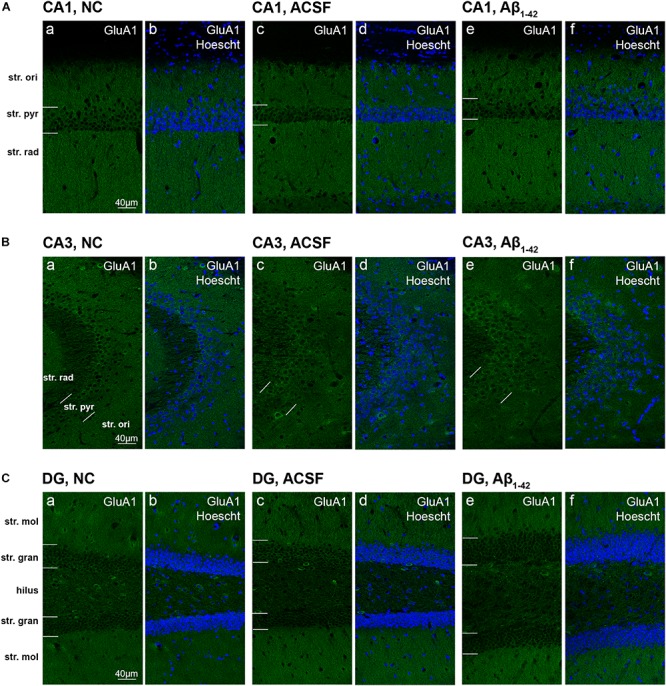
GluA1 expression in the mouse hippocampus 3 days post-injection. **(A–C)** Representative confocal images show GluA1 (green) and Hoescht (blue) immunofluorescence for NC **(a,b)**, ACSF-injected **(c,d)**, and Aβ_1–42_-injected mice **(e,f)** in the CA1 **(A)**, CA3 **(B)**, and DG **(C)** regions of the hippocampus. Scale bars = 40 μm.

**FIGURE 3:**
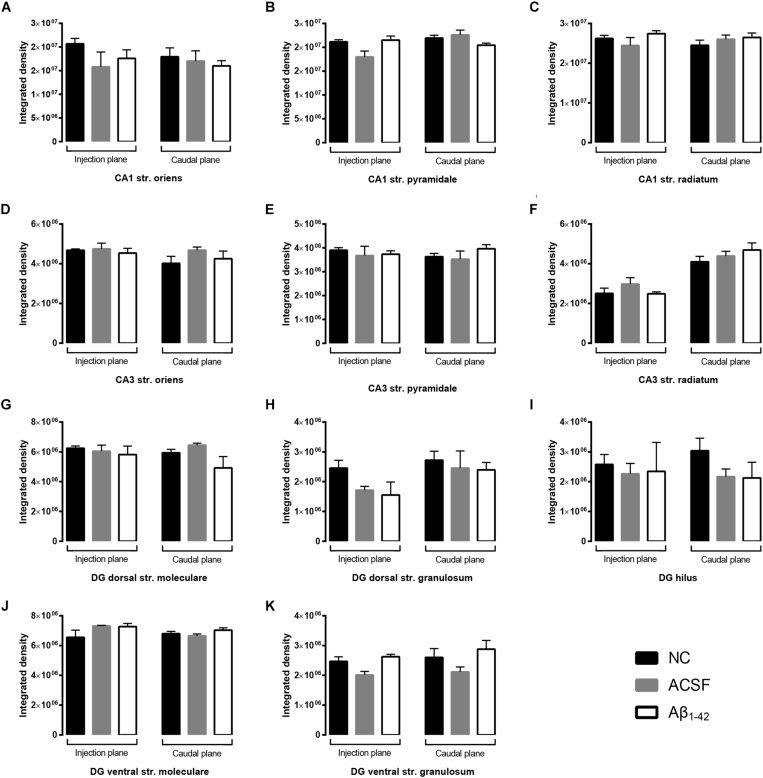
Aβ_1–42_ injection does not alter levels of GluA1 expression in the mouse hippocampus 3 days post-injection. **(A–K)** Graphs show quantification of GluA1 optical density in the str. oriens (str. ori), str. pyramidale (str. pyr), and str. radiatum (str. rad) of the CA1 and CA3 regions, and the hilus, str. moleculare (str. mol), and str. granulosum (str. gran) of the DG region. Data are expressed as mean ± SEM (Unpaired Mann–Whitney test; *n* = 6 NC, 6 ACSF injected mice and 6 Aβ_1–42_-injected mice). NC, naïve control; ACSF, ACSF-injected; Aβ_1–42_, Aβ_1–42_-injected mice.

The GluA1 receptor subunit displayed diffuse staining within the str. radiatum and str. oriens, with marked immunoreactivity localized to cellular processes within the str. pyramidale of the CA3 (Figure 2). Isolated localization to pyramidal cell bodies can be seen through all three layers of the CA3, although mainly concentrated within the str. pyramidale. The CA1 showed strong dense immunoreactivity within the str. oriens and str. radiatum, with relatively decreased staining within the str. pyramidale cells. Within the DG, immunoreactivity was diffuse within the str. moleculare, with staining localized to cellular bodies within the str. granulosum. In particular, the hilus displayed neuronal body staining, with otherwise weak diffuse immunoreactivity. There were no significant expression changes in GluA1 receptor subunit in any of the treatment groups compared to control in all three hippocampal regions analyzed (Figures 3A–K).

**FIGURE 4:**
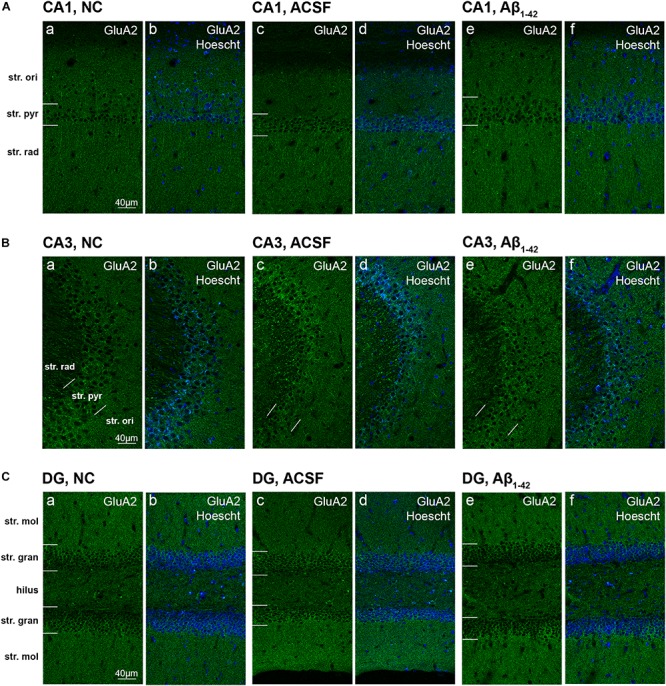
GluA2 expression in the mouse hippocampus 3 days post-injection. **(A–C)** Representative confocal images show GluA2 (green) and Hoescht (blue) immunofluorescence for NC **(a,b)** ACSF-injected **(c,d)** and Aβ_1–42_-injected mice **(e,f)** in the CA1 **(A)**, CA3 **(B)**, and DG **(C)** regions of the hippocampus. Scale bars = 40 μm.

**FIGURE 5:**
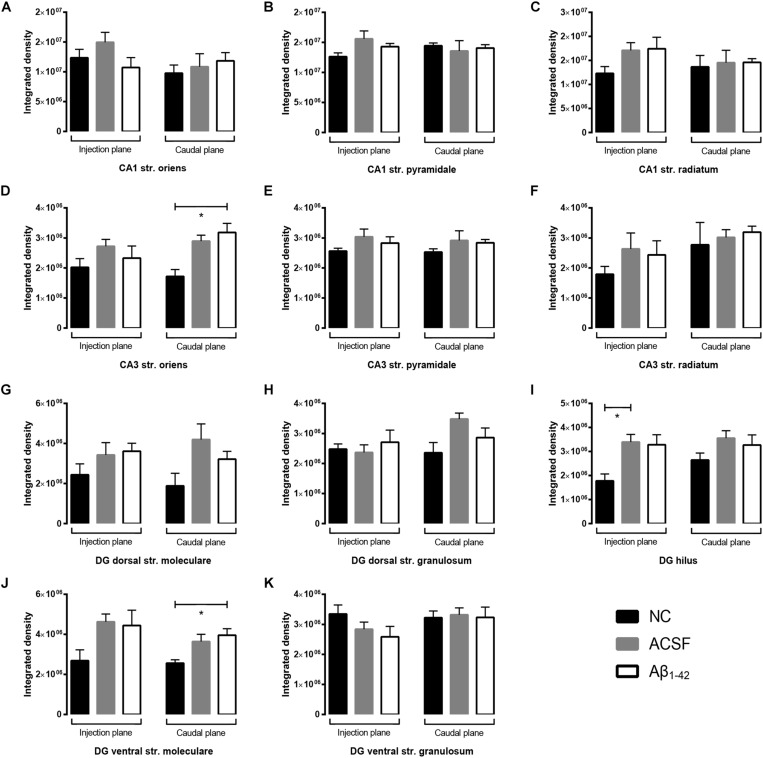
Aβ_1–42_ injected mice show altered hippocampal GluA2 expression within the CA1 when compared to NC mice. **(A–K)** Graphs show quantification of GluA2 optical density in the str. oriens (str. ori), str. pyramidale (str. pyr), and str. radiatum (str. rad) of the CA1 and CA3 regions, and the hilus, str. moleculare (str. mol), and str. granulosum (str. gran) of the DG region. Data are expressed as mean ± SEM (^∗^*p* < 0.05, Unpaired Mann–Whitney test; *n* = 6 NC, 6 ACSF injected mice and 6 Aβ_1–42_-injected mice). NC, naïve control; ACSF, ACSF-injected; Aβ_1–42_, Aβ_1–42_-injected mice.

GluA2 showed diffuse uniform staining within the str. radiatum and str. oriens of the CA3, with greater localization to neuronal bodies within the str. pyramidale (Figures 4Ba–f). The CA1 region exhibited similar staining patterns, localized to the cell bodies within the str. pyramidale, with diffuse staining throughout the str. oriens and str. radiatum (Figure 4A). In addition, immunoreactivity was localized to dendritic processes within the str. radiatum. Immunoreactivity within the DG was more diffuse within the str. moleculare, in contrast to the str. granulosum, which displayed more localized labeling surrounding cell bodies (Figure 4C). Labeling was also strong surrounding some neuronal cell bodies within the hilar region. There was a significant (*p* = 0.0400) increase in immunoreactivity of the GluA2 subunit within the injection plane of the DG hilus in ACSF-injected mice compared to control (Figure 5I). Increases in GuA2 subunit expression were also seen in the CA3 str. oriens (*p* = 0.0276) and DG ventral str. moleculare (*p* = 0.0236) in Aβ-injected mice when compared to naïve controls (Figures 5D,J). The ACSF-injected group showed the same trend of expression changes as the Aβ-injected group and there are no significantly different changes between these groups in any of the regions examined indicating an injection effect (Figures 5D,I,J). No other regions elicited any significant changes in GluA2 subunit expression between NC, ACSF-injected, and Aβ_1–42_-injected mice (Figures 5A–C,E–H,K).

### Expression of NMDA Receptor Subunits in the Hippocampal CA1, CA3, and Dentate Gyrus Regions

**FIGURE 6:**
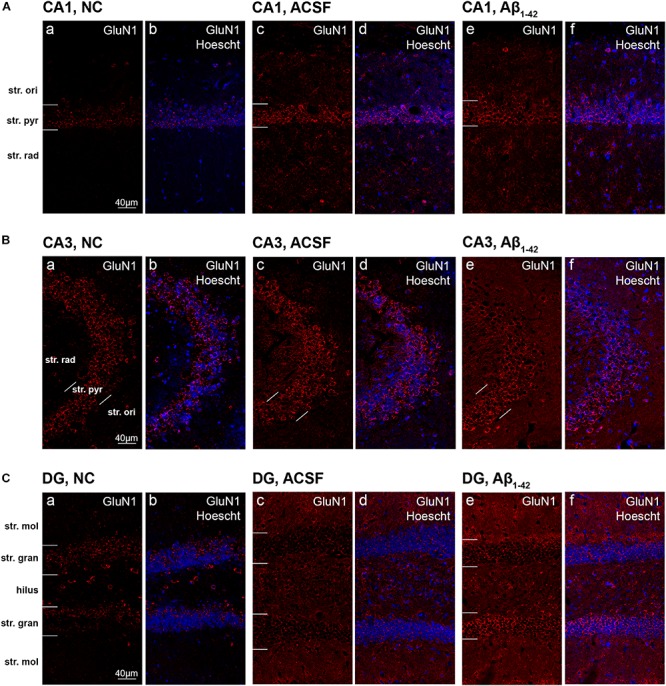
GluN1 expression in the mouse hippocampus 3 days post-injection. **(A–C)** Representative confocal images show GluN1 (red) and Hoescht (blue) immunofluorescence for NC **(a,b)**, ACSF-injected **(c,d)**, and Aβ_1–42_-injected mice **(e,f)** in the CA1 **(A)**, CA3 **(B)**, and DG **(C)** regions of the hippocampus. Scale bars = 40 μm.

**FIGURE 7:**
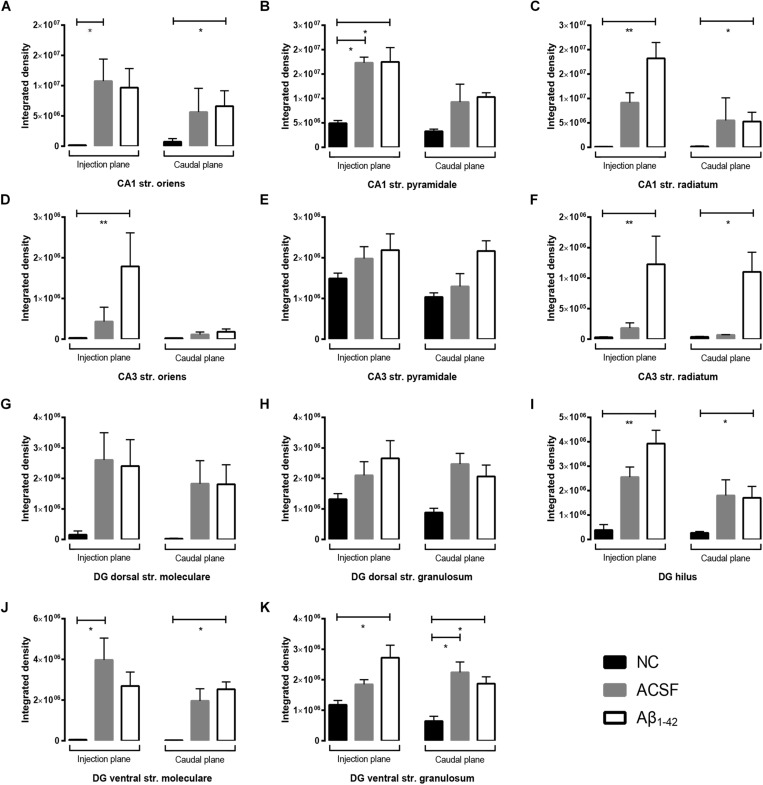
Aβ _1–42_ injection causes altered GluN1 expression within the mouse hippocampus 3 days post-injection. **(A–K)** Graphs show quantification of GluN1 optical density in the str. oriens (str. ori), str. pyramidale (str. pyr), and str. radiatum (str. rad) of the CA1 and CA3 regions, and the hilus, str. moleculare (str. mol), and str. granulosum (str. gran) of the DG region. Data are expressed as mean ± SEM (^∗^*p* < 0.05, ^∗∗^*p* < 0.01, Unpaired Mann–Whitney test; *n* = 6 NC, 6 ACSF-injected mice and 6 Aβ_1–42_-injected mice). NC, naïve control; ACSF, ACSF-injected.

GluN1 immunoreactivity appears localized to the membrane of str. pyramidale cells in the CA1, with reduced staining within the str. radiatum and str. oriens (Figure 6A). When compared to the NC group, immunoreactivity within Aβ-injected mice demonstrated much stronger immunoreactivity both at a diffuse level within the str. radiatum and str. oriens, as well as a stronger labeling surrounding cellular bodies which extends to some cells within the str. oriens (Figures 6Ac–f). Comparison between Aβ-injected and NC mice showed a statistically significant increase in expression within the caudal plane of the CA1 str. oriens (*p* = 0.0414) and str. radiatum (*p* = 0.0262) (Figures 7A,C), as well as the injection plane of the CA1 str. pyramidale (*p* = 0.0286) and str. radiatum (*p* = 0.0091) (Figures 7B,C). Increases were also seen within the ACSF-injected group compared to NC group within the injection plane of the str. oriens (*p* = 0.0216) and str. pyramidale (*p* = 0.0286) (Figures 7A,B). Overall, this indicates an increase in immunoreactivity of GluN1 subunits within all three layers of the CA1, particularly within the str. radiatum and the str. oriens. However, no significant differences in immunoreactivity were seen within any of the three layers of the CA1 between ACSF-injected and Aβ-injected mice. Expression changes within the str. oriens and str. pyramidale were responses to a microinjection stimulus, while in the str. radiatum GluN1 expression showed a trend toward increased expression in Aβ-injected mice compared to the ACSF-injected mice (Figure 7C).

GluN1 immunoreactivity within the CA3 followed a similar distribution, with staining strongest within the str. pyramidale, and limited punctate staining within the str. oriens and radiatum (Figure 6B). Results show a significant increase in expression of GluN1 receptor subunits within the injection plane of the str. oriens (*p* = 0.0037) and both injection (*p* = 0.0033) and caudal planes (*p* = 0.0148) of the str. radiatum in the CA3 in Aβ-injected mice compared to NC mice (Figures 7D,F). A similar trend in GluN1 expression was observed between Aβ-injected and ACSF-injected mice, although this did not reach statistical significance. Immunolabeling within the str. pyramidale appeared similar between NC, ACSF-injected and Aβ-injected mice (Figure 7E).

In the DG region, GluN1 immunoreactivity in NC mice followed a similar pattern seen within CA1 and CA3, with specific cellular staining within the dorsal and ventral str. granulosum (Figure 6C). ACSF-injected and Aβ-injected mice, however, showed a much stronger immunostaining that had a more diffuse picture within the hilus and str. moleculare, whilst retaining the specific cellular staining within the str. granulosum layers seen in NC mice. In addition, Aβ-injected and ACSF-injected mice displayed increased neuronal staining within the hilar area. Quantification revealed an increase in immunoreactivity within both the injection and caudal plane of the hilus (injection *p* = 0.0090; caudal plane *p* = 0.0353) and ventral str. granulosum (injection plane *p* = 0.0154; caudal plane *p* = 0.0372) in Aβ-injected mice compared to NC mice (Figures 7I,K). Similarly, increases in immunoreactivity were observed within the caudal plane of the ventral str. moleculare (*p* = 0.0315) (Figure 7J), although this was not statistically significant within its dorsal counterpart (Figure 7G). These changes were induced by the microinjection stimulus but Aβ further increased the expression of GluN1 in the hilus and ventral str. granulosum in Aβ-injected mice compared to ACSF-injected mice, although this increase did not reach significance (Figures 7I,K). No significant changes in immunoreactivity levels were seen within the dorsal str. granulosum (Figure 7H).

**FIGURE 8:**
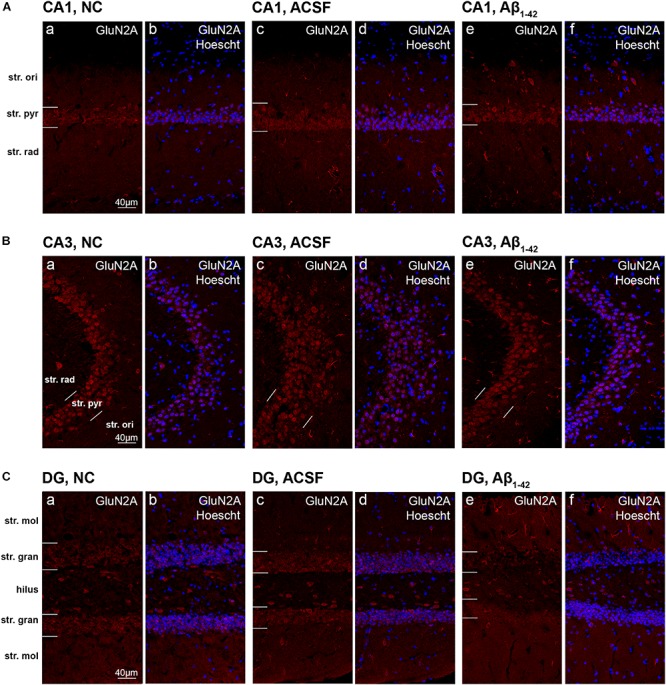
GluN2A expression in the mouse hippocampus 3 days post-injection. **(A–C)** Representative confocal images show GluN2A (red) and Hoescht (blue) immunofluorescence for NC **(a,b)**, ACSF-injected **(c,d)**, and Aβ_1–42_-injected mice **(e,f)** in the CA1 **(A)**, CA3 **(B)**, and DG **(C)** regions of the hippocampus. Scale bars = 40 μm.

**FIGURE 9:**
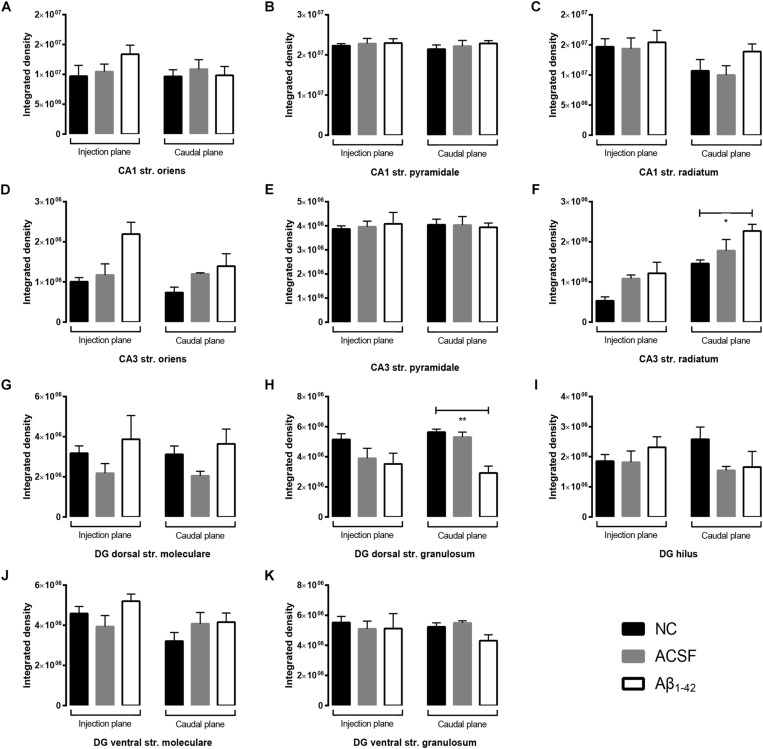
Aβ_1–42_ injected mice show altered hippocampal GluN2A expression within the CA3 and DG regions when compared to NC mice. **(A–K)** Graphs show quantification of GluN1 optical density in the str. oriens (str. ori), str. pyramidale (str. pyr), and str. radiatum (str. rad) of the CA1 and CA3 regions, and the hilus, str. moleculare (str. mol), and str. granulosum (str. gran) of the DG region. Data are expressed as mean ± SEM (^∗^*p* < 0.05, ^∗∗^*p* < 0.01, Unpaired Mann–Whitney test; *n* = 6 NC, 6 ACSF-injected mice and 6 Aβ_1–42_-injected mice). NC, naïve control; ACSF, ACSF-injected.

Similar to GluN1 immunoreactivity, the GluN2A receptor subunit was localized to the str. pyramidale of the CA1, however appeared to display increased labelling of the cell bodies in comparison to a more membrane-associated pattern seen in GluN1 immunostaining (Figure 8A). In addition, sparse dendritic-like staining could be observed within the str. pyramidale and str. radiatum. This was also observed within the CA3 region (Figure 8B), and within the str. granulosum layers of the DG (Figure 8C). The hilar region of the DG also exhibited strong cellular immunoreactivity. Some Aβ-injected specimens revealed specific localization of immunoreactivity to cellular bodies and their associated processes within the DG hilus and str. moleculare, with an increase in diffuse immunolabeling within the str. moleculare layers (Figure 8C). The densitometry analysis however showed that GluN2A staining remained largely robust within all layers of the CA1, CA3, and DG in NC, ACSF-injected, and Aβ-injected mice (Figures 9A–K). In contrast, a decrease in immunolabeling was detected (*p* = 0.0083) in the caudal plane of the DG dorsal str. granulosum in Aβ-injected mice compared to NC mice (Figure 9H). An increase (*p* = 0.0195) in immunoreactivity was also seen in the caudal plane of the CA3 str. radiatum in Aβ-injected mice compared to NC (Figure 9F). A similar trend in GluN2A expression was observed between Aβ-injected and ACSF-injected mice, although this did not reach statistical significance (Figures 9F,H).

### Expression of Vesicular Glutamate Transporters in the Hippocampal CA1, CA3, and Dentate Gyrus Regions

**FIGURE 10:**
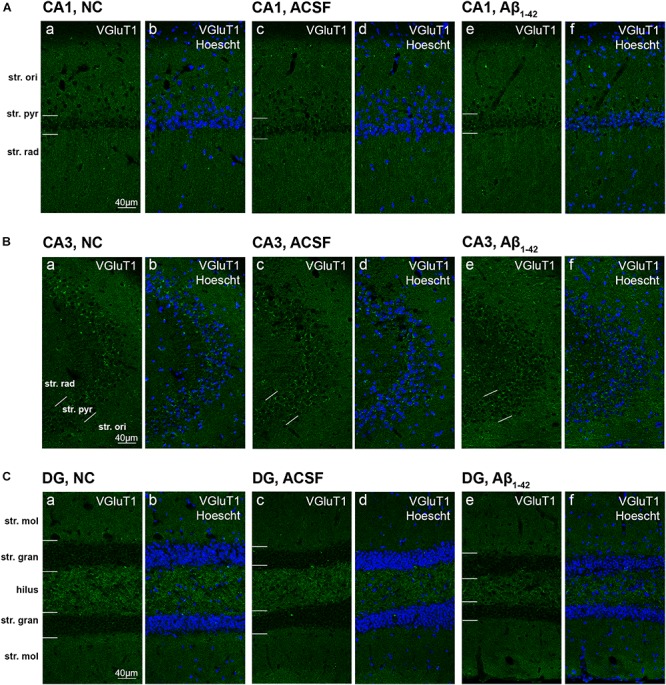
VGluT1 expression in the mouse hippocampus 3 days post-injection. **(A–C)** Representative confocal images show VGluT1 (gree) and Hoescht (blue) immunofluorescence for NC **(a,b)**, ACSF-injected **(c,d)**, and Aβ_1–42_-injected mice **(e,f)** in the CA1 **(A)**, CA3 **(B)**, and DG **(C)** regions of the hippocampus. Scale bars = 40 μm.

**FIGURE 11:**
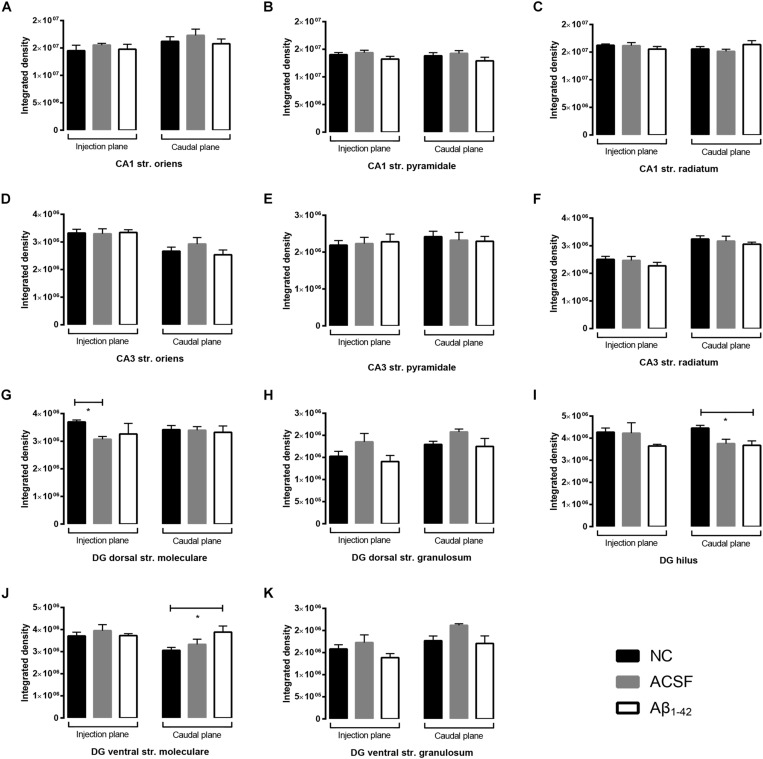
Aβ_1–42_ injected mice show altered hippocampal VGluT1 expression within the DG when compared to NC mice. **(A–K)** Graphs show quantification of VGluT1 optical density in the str. oriens (str. ori), str. pyramidale (str. pyr), and str. radiatum (str. rad) of the CA1 and CA3 regions, and the hilus, str. moleculare (str. mol), and str. granulosum (str. gran) of the DG region. Data are expressed as mean ± SEM (^∗^*p* < 0.05, Unpaired Mann–Whitney test; *n* = 6 NC, 6 ACSF injected mice and 6 Aβ_1–42_-injected mice). NC, naïve control; ACSF, ACSF-injected.

VGluT1 transporter staining in the CA1 is largely diffuse, with some faint localization to neuronal bodies particularly in the str. pyramidale (Figure 10A). VGluT1 immunoreactivity within the CA3 was mainly punctate within the str. radiatum and the str. pyramidale, with localization to cellular membranes within both the str. radiatum and str. oriens (Figure 10B). Expression of VGluT1 vesicular transporters appeared to be well-preserved in ACSF- and Aβ-injected mice 3 days post-injection within all three layers of the CA1 and CA3 regions (Figures 11A–F).

Immunoreactivity within the DG demonstrated a strong punctate staining within the hilus and a weaker staining was observed within the str. granulosum (Figure 10C). The str. moleculare exhibited a stronger diffuse staining than the str. granulosum, where staining was sparser and more localized to cellular membranes. Within the caudal plane of the DG, we found a significant increase (*p* = 0.0203) in VGluT1 expression within the ventral str. moleculare (Figure 11J), with a similar trend observed between Aβ-injected and ACSF-injected mice. We found a significant decrease (*p* = 0.0325) in GluN2A expression within the hilar region in Aβ-injected mice compared to NC (Figure 11I) and also observed a significant decrease (*p* = 0.0262) within the injection plane of the DG dorsal str. moleculare (Figure 11G) in ACSF-injected mice compared to NC mice; a similar trend was observed for the Aβ-injected group compared to NC mice, indicating an injection induced decrease. No significant changes in VGluT1 transporter expression was quantified in the dorsal and ventral str. granulosum layer (Figures 11H,K).

**FIGURE 12:**
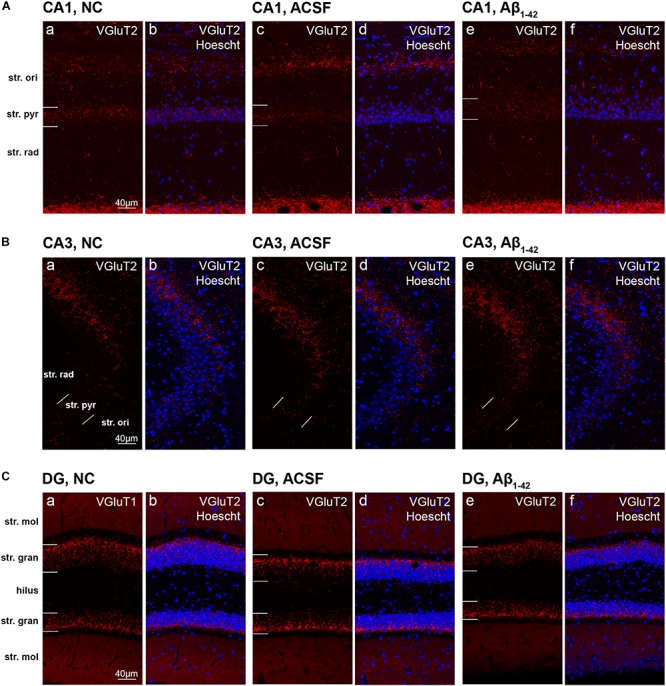
VGluT2 expression in the mouse hippocampus 3 days post-injection. (**A–C)** Representative confocal images show VGluT2 (red) and Hoescht (blue) immunofluorescence for NC **(a,b)**, ACSF-injected **(c,d)**, and Aβ_1–42_-injected mice **(e,f)** in the CA1 **(A)**, CA3 **(B)**, and DG **(C)** regions of the hippocampus. Scale bars = 40 μm.

**FIGURE 13:**
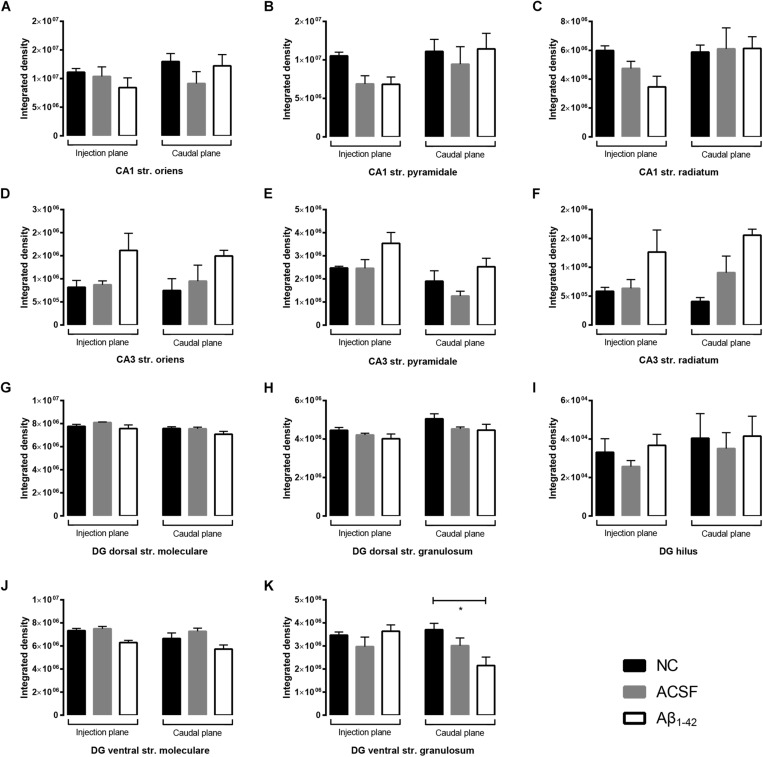
Aβ_1–42_ injected mice show altered hippocampal VGluT2 expression within the DG when compared to NC mice. **(A–K)** Graphs show quantification of VGluT2 optical density in the str. oriens (str. ori), str. pyramidale (str. pyr), and str. radiatum (str. rad) of the CA1 and CA3 regions, and the hilus, str. moleculare (str. mol), and str. granulosum (str. gran) of the DG region. Data are expressed as mean ± SEM (^∗^*p* < 0.05, Unpaired Mann–Whitney test; *n* = 6 NC, 6 ACSF injected mice and 6 Aβ_1–42_-injected mice). NC, naïve control; ACSF, ACSF-injected.

The VGluT2 transporter displayed similar punctate staining within the CA1 and CA3, however, staining was localized to the str. pyramidale, with reduced reactivity within the str. oriens and str. radiatum which do not appear to be associated with cellular bodies (Figures 12A,B). We report no VGluT2 transporter protein expression changes within the CA1 and CA3 regions between NC, ACSF-injected, and Aβ-injected mice (Figures 13A–F). In contrast to VGluT1 expression patterns, there was an absence of VGluT2 immunolabeling within the hilar region of the DG (Figure 12C). VGluT2 immunoreactivity was punctate within the str. granulosum, localized to cellular membranes, whilst the str. moleculare demonstrated much more diffuse staining with a lack of punctate reactivity. We found a significant decrease (*p* = 0.0298) in VGluT2 expression within the ventral str. granulosum of the DG in Aβ-injected mice compared to NC (Figure 13K) and a similar trend was observed between Aβ-injected and ACSF-injected mice. In all other layers of the DG, we did not detect any significant changes in VGluT2 expression between NC, ACSF-injected, and Aβ-injected mice (Figures 13G–J).

### Aβ_1–42_-Induced Cognitive Changes at Day 3 Post-injection

**FIGURE 14:**
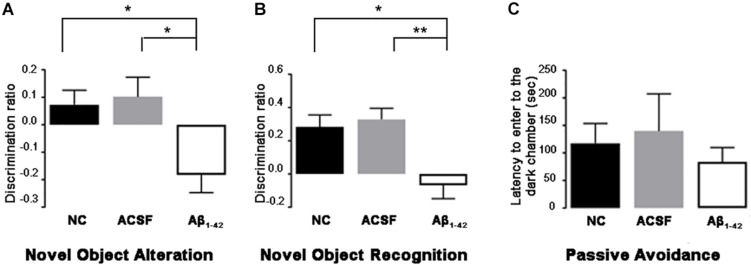
Aβ_1–42_-injected mice showed long-term spatial memory impairment revealed by the novel object alteration **(A)**, novel object recognition **(B)** tests. Non-spatial memory remained unchanged in Aβ_1–42_-injected mice compared with controls **(C)**. Data are expressed as mean ± SEM (one-way ANOVA, Bonferroni’s *post hoc* test) (^∗^*p* < 0.05; ^∗∗^*p* < 0.01, *n* = 12).

To elucidate the effect of Aβ**_1–42_** treatment on cognitive function the NOALT and NORT tests for long-term spatial-memory, and passive avoidance test for non-spatial memory were performed (Figure 14).

At day 3 post-injection, Aβ_1–42_-injected mice demonstrated significant spatial memory impairment compared with ACSF-injected (*p* = 0.0098) and NC mice (*p* = 0.0350) in the NOALT (Figure 14A). The significantly lower DR found in Aβ_1–42_-injected mice compared with ACSF-injected and NC mice indicated that Aβ_1–42_-injected mice could not detect changes in the location of object that had been moved.

At day 3 post-injection, Aβ_1–42_-injected mice showed significant spatial memory impairment compared with ACSF-injected (*p* = 0.0082) and NC (*p* = 0.0399) mice in the NORT (Figure 14B). The significantly lower DR found in Aβ_1–42_-injected mice compared with ACSF-injected and NC mice, indicates that Aβ_1–42_-injected mice could not discriminate between familiar and novel objects.

Aβ_1–42_-injected mice showed no significant difference in non-spatial memory performance when compared with the ACSF-injected and NC mice (Figure 14C). In phase 3 of the passive avoidance test (post-shock 3 h), similar latency (time taken to enter the dark chamber) was found in the control and Aβ_1–42_-injected mice (Figure 14C).

## Discussion

Although glutamatergic dysfunction has been reported in chronic neurodegenerative conditions such as AD, the precise effect Aβ has within the hippocampal environment is not well-understood. Previous studies have investigated effects of Aβ on neuronal conduction and excitability, but these mainly involve *in vitro* cell cultures. In addition, previous studies have not examined the localization and neuroanatomical expression of glutamatergic components in relation to specific regional and cell layers within the hippocampus. The present study demonstrates the effect of Aβ-injection on components of the glutamatergic system within specific regions and cell layers of the mouse hippocampus 3 days post-injection. Importantly, it also serves to quantify these effects on the glutamatergic system, and animal’s behavior in response to acute exposure to Aβ in an *in vivo* setting.

### AMPA Receptor Expression Alterations 3 Days Post-Aβ Injection

The GluA1 receptor subunit demonstrated robust expression patterns in the acute setting post Aβ-injection. Previous studies have indicated a loss in GluA1 expression post-Aβ exposure, secondary to a loss of scaffolding proteins at the post-synaptic membrane due to a variety of Aβ-mediated processes. Application of Aβ_1__–__40_ to cortical primary neurons and neuronal cultures resulted in a decrease in PSD-95, GluA1 and GluA2 (Almeida et al., 2005; Roselli et al., 2005). From this, it is postulated that degradation of PSD-95 as a result of Aβ application results in a concomitant decline in GluA1 expression. We were, however, unable to demonstrate loss of either GluA1 or GluA2, implying that the processes involved are a result of either chronic changes, or changes that only occur acutely in the artificial culture environment. A study by Zhang et al. (2018) demonstrated AMPAR internalization in human cultured primary neurons after application of Aβ, which was associated with an increase in AMPAR ubiquination (Zhang et al., 2018). This study involved Aβ treatment for 4 h, and whilst this represents an acute neuronal response, the *in vitro* nature of the experiment makes it difficult to extrapolate this to the physiological microenvironment of the brain. Similarly, Hsieh and colleagues demonstrated a loss of surface GluA1 and GluA2 after expression of β-CTF, the penultimate precursor of Aβ, at 22 h in CA1 hippocampal slices (Hsieh et al., 2006). This experimental design is still very limited in its capacity to represent acute neurotoxic effects seen in the cerebral setting.

Studies involving transgenic mouse models have also yielded similar results, reporting decreased GluA1 expression or a reduction in AMPA currents in mice overexpressing APP (Hsia et al., 1999; Almeida et al., 2005; d’Amelio et al., 2011), but transgenic models seek to replicate the chronic changes seen with clinical disease, thus does not offer any indication of potential acute changes. However in another study, Whitcomb et al. (2015) demonstrated application of oligomerized Aβ induced a rapid increase in AMPAR-mediated synaptic transmission 30 min after Aβ exposure, with an associated increase in surface expression of GluA1 in biotinylated hippocampal slices as rapidly, with no change in GluA2/3 expression.

While current literature indicates Aβ is involved in the downregulation of AMPARs and NMDARs, our results and results from Whitcomb et al. (2015) suggest another role of Aβ in the acute setting. Whitcomb et al. (2015) demonstrated amelioration of Aβ effects through inhibition of CaMKII, postulating a novel interaction between Aβ and CaMKII and PKA. CaMKII and PKA mediate phosphorylation, insertion, and synaptic stabilization of AMPARs (Opazo et al., 2012). As such, it is possible that in early disease, Aβ acts at normal physiological levels to stabilize and increase GluA1 receptor subunit expression at synaptic sites through potentiation and interaction with intrinsic molecules such as CaMKII and PKA. Our study demonstrates a timepoint later than studies reporting increased GluA1 expression and prior to studies showing decreased GluA1 expression, potentially indicating a chronological biphasic response to Aβ. Possibly, a rapid increase in AMPAR expression could present as an instantaneous acute response to neurotoxic exposure, which is followed by a secondary chronic phase resulting in reduction of AMPAR surface expression through a series of Aβ-driven mechanisms, including and not limited to ubiquination, dephosphorylation, and endocytosis. As such, our finding of no expression changes could be due to either no alterations in the early acute stages of Aβ administration, or a timepoint where dynamic expression changes have equilibrated.

### NMDA Receptor Expression Alterations 3 Days Post-Aβ Injection

There have been many studies characterizing the effect of acute Aβ administration on NMDAR-mediated currents (Domingues et al., 2007; Alberdi et al., 2010; Mezler et al., 2012). Our findings indicate varying degrees of increased GluN1 receptor subunit expression particularly within different layers of the CA3 region of the mouse hippocampus 3 days post-Aβ injection. In addition, alterations in expression were seen between ACSF-injected and NC mice. Similar to results seen in the AMPAR subunits, this was largely unexpected, as most current literature indicate Aβ’s primary inhibitory effect is on synaptic activity, in addition to its role in increasing ubiquination and internalization of NMDARs (Snyder et al., 2005). The NMDAR GluN1 subunit is an essential component of all functional NMDARs, therefore its homogenous expression can be used as a proxy for the number of NMDARs expressed at synaptic sites.

Currently, literature is still conflicting on Aβ’s effect on NMDAR activity, with some studies indicating Aβ-mediated aberrant activation of NMDARs resulting in increasing concentrations of cytosolic Ca^2+^ (Texidó et al., 2011), whilst others demonstrate Aβ-mediated selective inhibition of NMDAR activity (Zhang et al., 2009). This may be due to the different Aβ fragments used, or other experimental parameters present, in these studies (Zhang et al., 2009; Texidó et al., 2011).

Cullen et al. (1996) demonstrated reduced NMDAR synaptic transmission in the rat hippocampus more than 24 and 48 h after being intracerebroventricularly injected with Aβ, postulating that the delayed reduction in glutamatergic function may be due to an initial over-activation of NMDAR mediated synaptic transmission, reflecting a potentially biphasic response. As functional changes are only seen 24 h after Aβ exposure, expression changes, which involve more complex cellular pathways, may take a longer period to occur. In keeping with this hypothesis, Aβ_1–42_ intrahippocampal injection has been associated with a relative increase in GluN1 mRNA and protein expression 10 days post-injection compared to control mice, the extent of expression increase correlated in a dose-dependent manner (Peng et al., 2017). Our results indicate that such changes occur much earlier and can be evident 3 days post-injection. As a result, our anatomical findings of increased GluN1 subunit expression may be what follows immediately from the acute functional excitatory response, and may occur prior to the delayed reduction in AMPAR surface expression noted in other studies (Dewar et al., 1991; Chang et al., 2006; Hardt et al., 2014; Guntupalli et al., 2016). Furthermore, as illustrated prior, many studies involve *in vitro* experimentation, which does not take into account possible *in vivo* physiological mechanisms which may be neuroprotective and prevent NMDAR expression reduction in the acute setting. Studies demonstrating reduced NMDAR surface levels with acute (up to 3 days) exposure to Aβ have all been performed *in vitro* in hippocampal slice neurons and primary cortical neurons (Snyder et al., 2005; Hsieh et al., 2006).

The two most rigorously studied NMDAR subunits include the GluN2A and GluN2B, which have been implicated in disease processes (Ferreira et al., 2012; Tackenberg et al., 2013). The expression of these subunits dictate receptor function, and also the receptor’s response to physiological insults, such as exposure to toxic Aβ. For example, Aβ initiated GluN2B-containing NMDAR activation is able to suppress GluN2A-containing NMDAR activity (Liu et al., 2010). Despite literature suggesting significant disruptions to NMDAR composition and activity with Aβ interaction, our findings have demonstrated insubstantial changes in GluN2A expression in response to acute injection of Aβ.

### VGluT Expression Alterations 3 Days Post-Aβ Injection

Our findings indicate only minor changes in VGluT1 and VGluT2 transporter expression within the mouse hippocampus, although overall the transporters appear relatively robust after acute exposure to Aβ. While studies have identified the VGluTs as being preferentially affected in amyloidopathies such as AD, there is a lack of research examining acute Aβ effects on this transporter system. Studies have demonstrated a preferential accumulation of Aβ in glutamatergic neurons, with increased Aβ within synaptosomes co-labeled with both VGluT1 and Aβ (Sokolow et al., 2010). This study, however, does not examine the cause for this accumulation, and does not offer any insight into the potential mechanisms involved in this change. This accumulation of Aβ in AD has been shown to result in selective decline in VGluT1 expression (Rodriguez-Perdigon et al., 2016). Rodríguez-Moreno and Lerma (1998) noted a reduction in both glutamatergic terminals and VGluT1 levels in hippocampal cell cultures exposed to Aβ, with intracerebroventricular administration of Aβ_1–42_ resulting in altered synaptic plasticity and neuroinflammation.

On examining the Aβ-injection effect, Canas et al. (2014) demonstrated a preferential decrease in density of both VGluT1 and VGluT2 transporters in mice 15 days post-Aβ administration. In support of this, mice expressing the apoE4 gene demonstrated a reduction in VGluT1 levels in hippocampal neurons in conjunction with accumulation of Aβ and hyperphosphorylated tau (Liraz et al., 2013). This, however, represented a chronic accumulation of Aβ, which, while able to mimic possible chronic mechanisms and the pathological sequelae of apoE4 expression, is not able to be extrapolated to show the effect of acute Aβ insult on VGluT1 expression and function.

In our study there was only a minor increase in VGluT1 expression in the DG ventral str. moleculare and a decrease in VGluT2 expression in the str. granulosum in the Aβ-injected mice. This study demonstrates the robustness of the vesicular glutamate transport system, indicating changes noted in other studies are a result of longer more chronic exposure to Aβ.

The observed significant long-term spatial memory impairment is in line with studies conducted in the past examining the acute effect of Aβ injection on cognitive memory and function (Kim et al., 2016; Kasza et al., 2017). Mice treated with acute intracerebroventricular Aβ displayed statistically significant spatial memory impairment in Y maze test 3 days post-injection (Kim et al., 2016). Rats displayed impaired spatial memory on Morris water maze test and impaired synaptic plasticity 7 days post-intracerebroventricular Aβ_1–42_ injection (Kasza et al., 2017) but this timepoint might reflect more long-term consequences of the neurotoxic insult. We show no acute Aβ_1–42_-induced deficits in non-spatial memory performance and this is in line with findings in transgenic AD mouse models displaying these type of impairments only after extended periods of Aβ exposure. While the glutamatergic system is most likely involved in acute Aβ_1–42_-induced memory deficits, the robustness of the expression of receptor subunits and transporters indicate that other mechanisms might be involved which have to be further elucidated. Evaluating gene expression or other markers of glutamatergic signaling, e.g., proteins of the post-synaptic density, may be the focus of future research to deepen the knowledge into glutamatergic alterations by the Aβ protein and provide more information into disease mechanisms causing cognitive deficits.

## Conclusion

The results detailed in this study provide evidence on acute and focal effects of Aβ_1–42_ on memory function and the expression of components of the glutamatergic system in the mouse hippocampus. Importantly, although the glutamatergic system in early exposure is relatively robust against Aβ_1–42_-induced neurotoxic changes, even minor alterations in specific receptor subunit and transporter expression could lead to significant pathophysiological outcomes which is why glutamatergic changes in response to Aβ warrants further investigation.

## Data Availability Statement

All datasets generated for this study are included in the article.

## Ethics Statement

The animal study was reviewed and approved by the University of Otago and the University of Auckland Animal Ethics Committees.

## Author Contributions

JY, TP, WT, KP, and AK performed the research. AK, WT, RF, and HW designed the research. JY, TP, WT, HW, RF, and AK wrote the manuscript.

## Conflict of Interest

The authors declare that the research was conducted in the absence of any commercial or financial relationships that could be construed as a potential conflict of interest.
